# Design of colon-targeted drug delivery of dexamethasone: Formulation and in vitro characterization of solid dispersions

**DOI:** 10.1016/j.heliyon.2024.e34212

**Published:** 2024-07-06

**Authors:** Ahlam Zaid Alkilani, Sara Omar, Jehad Nasereddin, Rania Hamed, Rana Obeidat

**Affiliations:** a Department of Pharmacy, Faculty of Pharmacy, Zarqa University, Zarqa, Jordan; b Department of Pharmacy, Faculty of Pharmacy, Al-Zaytoonah University of Jordan, Amman, Jordan; c Department of Pharmaceutics and Pharmaceutical Technology, Faculty of Pharmacy, The University of Jordan, Amman, Jordan

**Keywords:** Dexamethasone, Solid dispersion, HPMC, Polymer, Eudragit S 100, Colon drug delivery

## Abstract

Colon-targeted drug delivery continues to generate increasing attention for its prospects in treating inflammatory bowel disease (IBD). This study aimed to develop and evaluate colon-targeted solid dispersions of dexamethasone (DEX-SDs) in vitro to reduce its systemic exposure. This would ultimately improve the therapeutic efficacy of DEX while minimizing its adverse effects. Different DEX-SDs formulations were prepared utilizing Eudragit S100 (EU S100) and a combination of hydroxypropyl methyl cellulose (HPMC) and EU S100 to tune its drug release profile suitable for colonic delivery. The fabricated formulations were extensively characterized via Attenuated Total Reflectance – Fourier Transform Infrared Spectroscopy (ATR-FTIR), differential scanning calorimetry (DSC), powder X-ray diffraction (PXRD), and polarized light microscopy (PLM). The different characterization techniques strongly suggest preparing solid solution-type solid dispersions of DEX with the other polymers (DEX-SDs). In addition, the in vitro dissolution of DEX-SDs was evaluated using two dissolution media (pH 1.2 and 7.4). The in vitro release of DEX-SDs was low in the acidic media and higher and sustained in the basic medium, leading to the conclusion that the developed DEX-SDs may represent an effective technology can overcome challenges related to poor drug solubility and bioavailability.

## Graphical abstract

**Figure:**
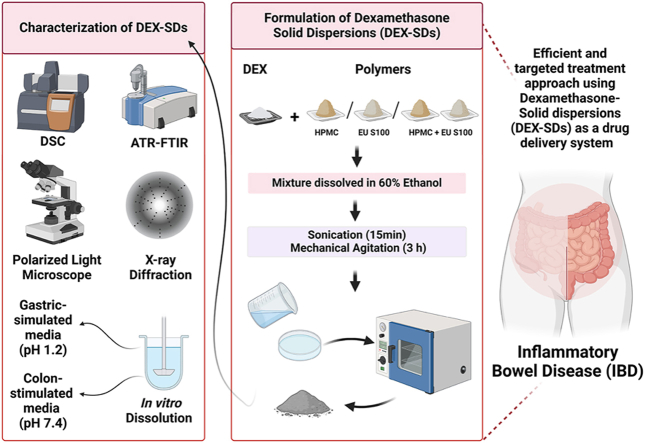

## 1 Introduction

Chronic conditions, including Crohn's disease and inflammatory bowel disease (IBD), are characterized by intestinal inflammation [1,2]. IBD prevalence in Western nations was 0.3 % in 2017, considered the highest prevalence. Additionally, it was shown that the prevalence of this condition is rising annually across the world [2,3]. IBD is currently treated with systemic drug delivery, which can result in side effects and decreased efficacy due to poor colonic drug exposure. To address these issues, there is a need to develop more efficient and targeted treatment approaches [4,5].

Several commercially available formulations that contain corticosteroids and immunomodulators have been used to treat IBD [6]. Corticosteroids (CSs) include cortisone, prednisone, budesonide, dexamethasone (DEX), and methylprednisolone used to treat IBD patients. They have a potent anti-inflammatory effect and are highly effective [7].

The purpose of the present study is to address the issues associated with the conventional delivery of DEX, particularly its limited bioavailability and adverse effects associated with systemic exposure. Dexamethasone (DEX) is a potent corticosteroid with an oral bioavailability of around 70 % [8]. The drug works by reducing inflammation in the digestive tract and suppressing the immune system. The drug is usually taken orally as tablets; the dose varies depending on the symptoms' severity. DEX has extensive side effects such as acne and adrenal insufficiency; visual changes may occur due to steroid-induced hyperglycemia, osteoporosis, and high blood pressure [9]. In addition, DEX might cause immunosuppression. Since it inhibits T cell proliferation and differentiation by suppressing the CD28 co-stimulatory pathway [10].

Solid dispersions (SDs) are drug delivery systems that incorporate the drug into a polymeric carrier to improve its physical and biopharmaceutical properties [11,12]. One of well-known brand names for itraconazole and valsartan are Sporanox® and Entresto®, respectively, which are an example of approved medications that utilize solid dispersion (SD) technology. These approved formulations not only highlights how advanced pharmaceutical technologies can overcome challenges associated with poor solubility and bioavailability but also ensures more consistent therapeutic effects and better patient compliance [[13], [14], [15]].

In colon-targeted SDs, the drug is incorporated into a polymeric matrix along with a colon-specific targeting agent. Traditionally, solid dispersions could utilize either crystalline or amorphous polymeric matrices [16]. However, extensive studies confirmed that the ability of drugs to dissolve within crystalline polymeric carriers is restricted [[17], [18], [19]] and that the term “solid dispersions” has been recently more formalized to exclusively refer to systems in which the drug is maintained in an amorphous state within the carrier matrix [20,21]. Polyvinyl pyrrolidine (PVP), Eudragit S100 (EU S100), and hydroxymethyl cellulose-AS (HPMC-AS) are examples of amorphous polymeric carriers that are commonly employed in solid dispersions [17,19,[22], [23], [24]].

This study aims to develop and evaluate colon-targeted dexamethasone solid dispersions (DEX-SDs) in vitro to reduce systemic exposure and minimize DEX side effects. The DEX-SDs formulations were designed to resist degradation in the stomach and small intestine and release the drug in the colon only using a pH-sensitive polymer such as Eudragit S100 [25]. The addition of Eudragit® S 100 in the formulation offers many advantages due to its excellent biocompatibility and suitability for colon-targeted drug delivery applications. This approach aimed to increase the local concentration of DEX in the colon while reducing its systemic exposure, improving its therapeutic efficacy, and minimizing DEX adverse effects such as hormonal disturbances, weight gain, mood changes, and, in chronic cases, Cushing's Syndrome [26,27]. However, there are also some challenges associated with the use of colon-targeted SDs. For instance, the stability of SDs over time and under different storage conditions is a cause of concern. It should be investigated thoroughly, particularly in situations where the SD formulation is only kinetically stabilized (via immobilization of the drug), as opposed to thermodynamically stabilized solid dispersions (systems in which the drug is molecularly dispersed and intimately bonded with the polymeric carrier) [28,29].

DEX-SDs were characterized for drug loading efficiency and yield, Fourier-transform infrared spectroscopy (FTIR), Differential Scanning Calorimetry (DSC), Powder X-ray diffraction (PXRD), polarized light microscope (PLM), solubility, dissolution, and mechanism of drug release. The results of this study may have the potential to provide a new and effective treatment approach for IBD and enhance our understanding of the use of solid dispersions for targeted drug delivery.

## 2 Materials and methods

### 2.1 Materials

Dexamethasone (DEX) was kindly supplied by Angene Chemical (Nanjing, China). EU S100 was obtained from Corel Pharma Chem. (Gujarat, India). HPMC was purchased from Sigma-Aldrich (Dorset, UK). Acetonitrile, methanol, and ethanol (HPLC grade) were obtained from Tedia (Fairfield, OH, USA). All other reagents and chemicals used in this research were of analytical grade.

### 2.2 Preparation of dexamethasone solid dispersions (DEX-SDs)

Dexamethasone solid dispersions (DEX-SDs) were prepared by the solvent evaporation method [12]. EU S100 and a combination of HPMC and EU S100 at different ratios were used to prepare DEX-SDs. The ratio of DEX-to-polymers was 5:95 % (w/w). The drug and polymers were dissolved separately in 100 mL of 60 % ethanol, which was used as a solvent. Then, the drug and polymer solutions were mixed together under mechanical agitation for 3 h, sonicated for 15 min, and placed in an oven maintained at 40 °C to evaporate the solvent and form a solid mass completely. The solid mass was then crushed into powder and stored in a desiccator until further use. The composition of DEX-SDs is shown in **Table (1).**

### 2.3 Drug loading efficiency and yield

The drug loading efficiency (DLE) and yield of DEX-SDs were determined using high-performance liquid chromatography (HPLC). A sample of 435 mg of DEX-SDs containing 20 mg DEX was prepared to achieve a concentration of 500 μg/mL. The concentration was then diluted twice and injected into the HPLC unit in triplicate. The area under the peak was determined and used to calculate the drug loading efficiency and yield using Equations Equation (1), Equation (2) [30].

Equation (1) $$DLE\;\%=\frac{\text{Amount}\;\text{of}\;\text{drug}\;\text{measured}}{\text{Theoretical}\;\text{amount}\;\text{of}\;\text{drug}\;\text{based}\;\text{on}\;\text{drug}\;\text{loading}\;}*100\%$$

Equation (2) $$\text{Yield}\;\%=\frac{\text{Weight}\;\text{of}\;\text{prepared}\;\text{solid}\;\text{dispersion}}{\text{Weight}\;\text{of}\;\text{drug}\;‏\;\text{carrier}}*100\%$$

### 2.4 High-performance liquid chromatography (HPLC) method

Separation was carried out using HPLC (Shimadzu LC-20AT Pump, Standard Autosampler, SPD-20A UV/VIS Detector, Shimadzu, Kyoto, Japan) on C18 column (4.6 mm × 15 cm) (Phenomenex, Torrance, USA) using a mixture of water: acetonitrile (70:30). The mobile phase was set at a flow rate of 1 mL/min. For HPLC analysis, all injection volumes were 20 μL. UV detection was performed at a wavelength of 245 nm. The DEX peak was detected at a retention time of 8.5 min. Methanol was used as a solvent to dissolve DEX at a drug concentration range of 10–150 μg/mL to construct a standard calibration curve of DEX (R^2^ = 0.999).

### 2.5 Attenuated total reflection-Fourier-transform infrared spectroscopy (ATR-FTIR)

Samples of pure DEX, HPMC, EU S100, and DEX-SDs were ground and analyzed. The FTIR spectra were acquired in absorbance mode using a PerkinElmer UATR-II device (Waltham, MA, USA) using a wavelength range of 4000 cm^−1^ – 550 cm^−1^ with a scanning resolution of 4 cm^−1^ and 32 repeated scans at room temperature. The acquired spectra were then exported in Comma Separated Values (CSV) format and analyzed using Ira FTIR Data Explorer Build #210223 (Zarqa University, Zarqa, Jordan) [31].

### 2.6 Differential scanning calorimeter (DSC)

The DSC analysis of pure DEX, polymers, and DEX-SDs and their corresponding physical mixtures was performed using (DSC-50 Q Shimadzu, Japan) equipped with a Shimadzu TA-50 WSI instrument controller and a Shimadzu professional computer. Samples were placed in aluminum pans, and a reference scan was performed using an empty aluminum pan. Before the actual scanning process, any residual moisture in the samples was removed by pre-heating them to a temperature of about 25–40 °C, below the first glass transition for 30 min. The dried samples were then scanned from 25 to 300 °C at a heating rate of 5 °C/min and a 50 mL/min nitrogen gas flow.

### 2.7 Powder X-Ray diffraction

Powder X-Ray diffraction (PXRD) patterns of pure DEX, polymers, and DEX-SDs and their corresponding physical mixtures were acquired using an Ultima IV X-ray diffractometer (Rigaku, Japan) equipped with a cobalt radiator at a voltage of 40 KV and a current of 30 mA. The angle (2 θ) scanning range of the samples was between 0° and 60° at a step of 0.02°.

### 2.8 Polarized light microscope (PLM)

DEX-SDs were identified using a polarized light microscope (PLM). An optical microscope (Bausch and Lomb Medical Microscopes, Javal, Japan) equipped with polarizing filters and a digital camera was used to observe the presence of amorphous material at room temperature. About 1 mg of each sample was placed on glass slides, covered with glass covers, and viewed under a 10x lens magnification. Images were captured for each sample under the PLM using Deltapix software.

### *2.9* *In vitro* dissolution studies

#### 2.9.1 Dissolution study in acidic media (pH 1.2)

The release of DEX from SDs was investigated in a dissolution medium that mimics the gastric fluid with a pH of 1.2 [32]. The simulated gastric fluid was prepared with 0.1 M hydrochloric acid (HCl) and maintained at 37 °C with paddles rotating at 50 revolutions per minute (rpm). Samples containing 50 mg of DEX-SDs were placed in capsules in dissolution apparatus II (PTWS 620I, Copley®, UK) and exposed to the dissolution medium for 120 min. At specific time intervals (5, 10, 15, 30, 45, 60, 90, and 120 min), a volume of 3 mL was withdrawn. The withdrawn volume was replaced with an equal amount of fresh dissolution medium to ensure a consistent concentration gradient. The samples were then analyzed using the HPLC method at a wavelength of 254 nm. Each experimental run was conducted in triplicate.

#### 2.9.2 Dissolution study in colon-stimulated media (pH 7.4)

The dissolution procedure was repeated for DEX-SDs in a colon-simulating medium, following their dissolution in the acidic medium (pH 1.2). In this case, a dissolution medium of 900 mL was prepared using 0.1 M phosphate buffer (pH 7.4), [33]. The temperature of the dissolution medium was maintained at 37 °C, and the paddle was rotated at 50 rpm. The phosphate buffer was composed of 0.075 M dibasic potassium phosphate (K_2_HPO_4_) and 0.025 M monobasic potassium phosphate (KH_2_PO_4_), according to a study by Carredano et al. (2018) [34]. For each dissolution vessel, 50 mg of DEX-SDs were introduced. Samples underwent dissolution for a period of 6 h. At specific time intervals (5, 10, 15, 30, 45, 60, 90, 120, 180, 240, 300, and 360 min), a volume of 5 mL was withdrawn. The withdrawn volume was replaced with an equal amount of fresh dissolution medium to maintain a consistent concentration gradient. Subsequently, the samples were filtered and subjected to analysis using the HPLC method.

### 2.10 Solubility study

The solubility of pure DEX and DEX-SDs was compared using the shake flask method [35]. The solubility of DEX was determined in two solvents: phosphate-buffered saline (PBS, pH = 7.4) and water. An excess amount of pure DEX and DEX-SD was added to 5 mL of PBS (pH = 7.4) or water in glass vials. The vials were placed in a shaker incubator, maintained at 37 ± 1 °C or 25 ± 1 °C, and agitated at 100 rpm for 24 h. Aliquots of 1 mL were taken from each sample, filtered through a 0.45 μm membrane, and appropriately diluted with PBS. The concentration of DEX was analyzed using the HPLC unit. All experiments were repeated three times, and the results were reported as mean ± SD.

### 2.11 Statistical analysis

All measurements were repeated, and the results were expressed as the mean ± standard deviation (SD). The data from the different groups were statistically analyzed using a one-way analysis of variance (ANOVA). P values less than 0.05 (*p* < 0.05) were considered statistically significant. Microsoft Office Excel application was used for the calculations.

## 3 Results and discussion

### 3.1 Preliminary study to prepare dexamethasone solid dispersions (DEX-SDs)

The solvent evaporation method is preferred for its advantages over other techniques. It is particularly valuable for formulating heat-sensitive drugs due to its low-temperature solvent evaporation process [36]. Additionally, the rapid solvent evaporation in this method results in amorphous drug-polymer dispersions, enhancing drug solubility and bioavailability [36,37]. The solvent evaporation method was used to fabricate solid dispersions (SDs) with different ratios of DEX and polymers, specifically EU S100 and HPMC. This method developed an obviously clear and transparent film. After that, the precipitate of formula was scratched from the watch glass and sliced with a sterilized razor blade to obtain power. However, it is significant to note that the final amount of powder obtained after scratching the film from the watch glass differs from the initial amount. This disparity resulted from a portion of the formula adhering to and remaining on the watch glass surface throughout the scratching process. Adhesion of cast polymeric films to fabrication surfaces is well-documented to be a function of the surface free energy and the ability of the polymer to interact and/or bond with the fabrication surface (a watch glass, in this case) [38].

Furthermore, polymer-surface interactivity has been reported to significantly affect the appearance of the fabricated dosage form, depending on the likelihood that the polymer may interact with the fabrication surface; a study by Al-Hijjaj and Nasereddin et al. investigated this effect on different fabrication surfaces (polyimide, aluminium, and glass) [39]. Detachment off the glass surface in particular was found easier when hydrophilic polymers were cast on the surface, and more challenging hydrophobic polymers were cast onto the plate [39]. The reported results corroborate what was observed herein, in which the freely water soluble HPMC detached off the build plate whole with no residual adherence to the build plate, while the formulas containing HPMC and EU S100 were more challenging to remove from the watch glass, which is likely due to the more hydrophobic acrylate EU S100 forming a stronger interaction with the glass.

**Fig. 1:**
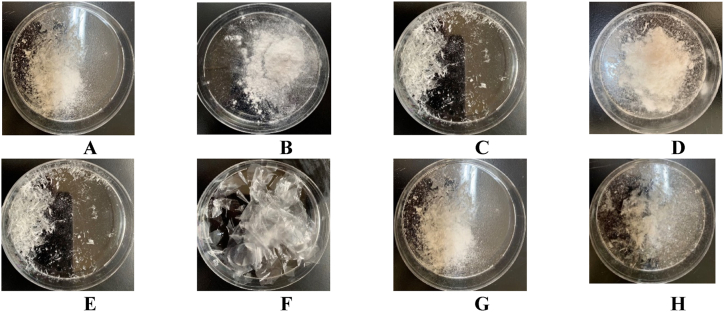
Preliminary study for solid dispersion formulations for A) SD1, B) SD2. C) SD3, D) SD4, E) SD5, F) SD6, G) SD7, and H) SD8.

We conducted a preliminary study to formulate SDs using different polymer ratios, as summarized in Fig. 1 (A-H). However, many of these formulations had to be excluded from further consideration due to concerns related to the developed film's adhesion properties and the remaining formula amount. Therefore, we formulated SDs as summarized in Table 1. Specifically, SD1 contains EU S100, while SD2 includes HPMC and EU S100.

**Table 1:** Composition of DEX-SDs using EU S100 and a combination of HPMC and EU S100. The total weight of DEX and polymers equals 500 mg.

Formulas no.	HPMC (w/w) %	EU S100 (w/w) %	EC (w/w) %	DEX (w/w) %
**SD1**	40 %	60 %	0 %	5 %
**SD2**	0 %	100 %	0 %	5 %
**SD3**	60 %	40 %	0 %	5 %
**SD4**	20 %	80 %	0 %	5 %
**SD5**	80 %	20 %	0 %	5 %
**SD6**	100 %	0 %	0 %	5 %
**SD7**	0 %	90 %	10 %	5 %
**SD8**	0 %	100 %	0 %	2 %

### 3.2 Drug loading efficiency and drug content

A 500 mg of the physical mixtures of DEX-SDs, containing 25 mg DEX and 475 mg polymers, have been prepared. The total actual amount of DEX-SDs was 435 mg; the remaining amount was lost in the watch glasses. DEX-SDs were successfully prepared using the solvent evaporation method with a relatively high yield of 86 %. In addition, the drug content was 92.06 ± 1.25 and 95.93 ± 0.45 % for SD1 and SD2, respectively.

### 3.3 Differential scanning calorimetry (DSC)

**Fig. 2:**
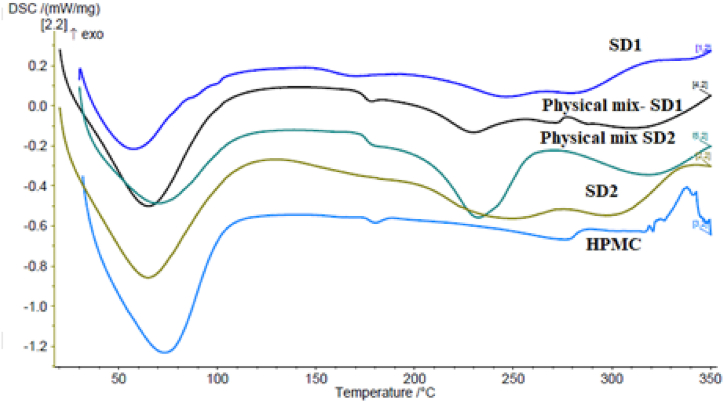
DSC thermograms of DEX, EU S100, HPMC, SD1, SD2, and the physical mixtures of SD1 and SD2.

Fig. 2 shows HPMC, SD1, and SD2's DSC thermograms and their corresponding physical mixtures. All thermograms exhibited a broad endothermic event around 80 °C, consistent with moisture loss. The DSC thermogram of HPMC showed an endothermic event at 165 °C, which likely corresponds to HPMC's glass transition temperature (Tg), manifesting as thermal relaxation and consistent with the commonly reported thermal behavior [40,41]. In the thermograms of the physical mixtures of SD1 and SD2, the two notable features are an endothermic event occurring at 240 °C, and a heat capacity change consistent with T_g_ observed at approximately 170 °C. The physical mixtures were further analyzed using a Heat-Cool-Reheat (HCH) DSC cycle [42], as shown in **(supplementary files)**. The first heating cycle of the HCH thermograms is identical to what was seen in the ramp program, with the moisture loss endotherm being seen at approximately 80 °C, followed by the presumed T_g_ seen at 170 °C, with the endothermic event being seen at 240 °C. In both thermograms, the cooling cycle showed no signs of recrystallization, with the only significant event seen being a heat capacity change consistent with T_g_ at approximately 130 °C. The thermogram of the second heating cycle were similarly unremarkable, barring the aforementioned T_g_ being seen at 130 °C. The thermograms of formulations SD1 and SD2 did not show any endothermic events consistent with melting (T_m_). A heat capacity change can be seen in the thermogram of SD1 at approximately 130 °C, while that of SD2 thermogram of SD2 is unremarkable.

Thermal dissolution (melting point depression) is a well-documented phenomenon in which drug crystals that are physically mixed with a compatible polymer that is maintained at above T_g_ begin to slowly dissolve into the polymer, which often manifests in DSC thermograms as an asymmetrical, broadened melting point [[43], [44], [45]]. The T_g_ of EU S100 is well documented to be approximately 180 °C [46,47]. Therefore, assuming drug/polymer compatibility, it is highly likely that the endothermic event seen at 240 °C corresponds to depressed T_m_ of DEX, which usually occurs at 262 °C [48], indicating the compatibility of DEX and EU S100. This is further supported by the absence of any melting endotherms in both the cooling cycle and second heating cycle, suggesting complete amorphization of DEX and the formation of a solid dispersion whose T_g_ lies at 130 °C, which was seen in both the cooling and second heating cycles, as well as in the SDs thermograms.

**Fig. 3:**
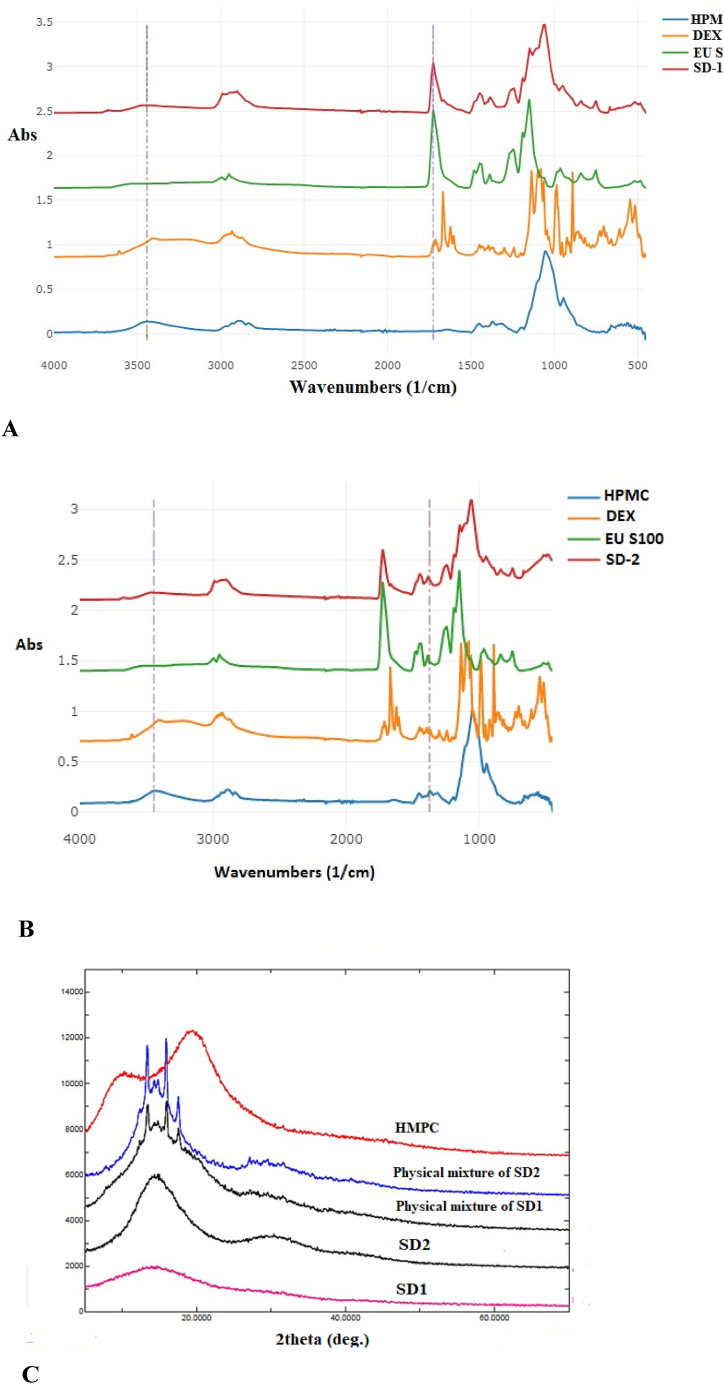
FTIR spectra of A) SD1 and its components and B) SD2 and its components C) Powder X-ray diffraction pattern of HMPC; physical mixture of SD2; physical mixture of SD1, SD2, and SD1.

When examined in the context of the FTIR spectra (Fig. 3), the observed melting point depression and the absence of any endothermic events in the second heating cycle of the physical mixtures and SD formulations further support the compatibility of DEX and EU S100 and the formation of a thermodynamically stabilized SDs. The FTIR spectra are presented and discussed extensively in the upcoming section.

### 3.4 FTIR

Fig. 3A and B shows the FTIR spectra of DEX, HPMC, EU S100, SD 1 and SD 2. The characteristic peaks of DEX were observed at 3408, 1706, and 1662 cm^−1^, corresponding to OH stretching and the two C=O stretching peaks of DEX, respectively [49]. Whereas the characteristic peaks of EU S100 were observed at 3224 cm^−1^ and a peak at 1726 cm^−1^ and 1705 cm^−1^ related to the carbonyl group, likely representing OH stretching and C=O stretching of the two carbonyl groups [50]. The characteristic peaks of HPMC were seen at 3452 cm^−1^, likely representing OH stretching, and at 1312 cm^−1^, likely representing OH bending peak [51]. In the FTIR spectrum of SD1, a peak was seen at 3443 cm^−1^, potentially corresponding to DEX's shifted OH stretching peak.

Additionally, the peak seen at 1723 cm^−1^ likely represents the shifted C=O stretching of EU S100. The observed shift in the OH and C=O groups of DEX and EU S100, respectively, suggests an intermolecular interaction between the drug and polymer matrix. This coupled with the disappearance of the melting endotherm of DEX (Fig. 2, Fig. 3A) in the DSC thermograms of the formulation, along with the absence of any crystallographic peaks and the appearance of the characteristic “amorphous halo” in the PXRD diffraction pattern (Fig. 3C) strongly suggests that the DEX/EUS100 interaction alluded to is an indication of a thermodynamically stabilized solid dispersion mediated by hydrogen bonding along the DEX OH groups and the EUS100 C=O groups.

### 3.5 Powder X-ray diffraction (PXRD)

**Fig. 4:**
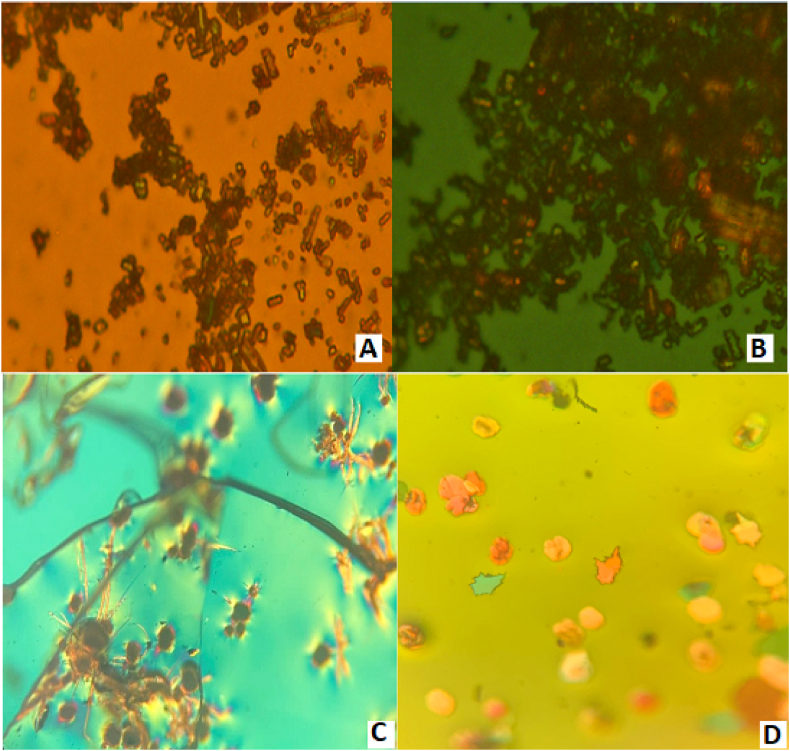
Polarized photographs of A) DEX, B) DEX (Dark background) C) SD1, and D) SD2 (Magnification 10×).

Fig. 3C shows the PXRD patterns of HPMC, DEX-SDs, and their corresponding physical mixtures. Because DEX is known to have a crystalline structure [52,53], it is noteworthy that we did not conduct a PXRD study on it. Two characteristic peaks at 8° and 20° on the HPMC diffractogram agree with the previous study [54]. The PXRD patterns of HPMC, SD1, and SD2 exhibited no visible diffraction patterns but displayed the characteristic amorphous halo [55,56]. However, the physical mixtures of SD1 and SD2 showed specific peaks at 2θ in the range of 5–20°, indicating a crystalline content from DEX, as previously described in literature [52,53]. In Fig. 4, the amorphous nature of DEX was observed in the PXRD patterns of SD1 and SD2 when compared with their corresponding physical mixtures. The PXRD results corroborated with the DSC thermograms, suggesting that SD1 and SD2 contain DEX in the molecularly dispersed phase with no crystalline form. Dong et al. [57] investigated the PXRD patterns for atorvastatin solid dispersions and their corresponding physical mixtures. When they examined the PXRD patterns of the physical mixtures, they noticed visible peaks at specific angles, which closely resembled those of the original drug. However, when they analyzed the PXRD patterns of the solid dispersions, no distinctive peaks corresponding to the bulk atorvastatin were noticed. This suggested that the drug was precipitated as an amorphous form, in agreement with our findings.

### 3.6 Polarized light microscopy (PLM)

PLM images were captured at room temperature to gain more insight into the physical state of the freshly prepared DEX-SDs. By observing the samples under polarized light, the presence or absence of birefringence can be used to distinguish between crystalline and amorphous forms of the drug [58]. Typically, the presence of birefringence suggested a crystallinity, while an amorphous form displayed no birefringence [58].

Fig. 4 presents the images of DEX and DEX-SDs. Birefringence was observed for pure DEX, indicating a crystalline form (Fig. 4A and B). In contrast, birefringence was seen in DEX-SDs (SD1 and SD2), suggesting the presence of some crystalline DEX, which was not detected in the PXRD patterns as shown in Fig. 4C and D. Overall, both systems are highly amorphous, while there is evidence for the existence of traces of crystals, as shown in PLM results. This agrees with Marano et al. [30], who studied the development of olanzapine and piroxicam solid dispersion. Birefringence was observed in piroxicam SDs but not in olanzapine SDs, indicating the presence of some crystalline substance that XRPD did not detect. When a solid dispersion formulation exhibits birefringent light diffraction, it could indicate the presence of some crystalline material, such as EU S100 or the used drug [59].

### 3.7 Dissolution study

**Fig. 5:**
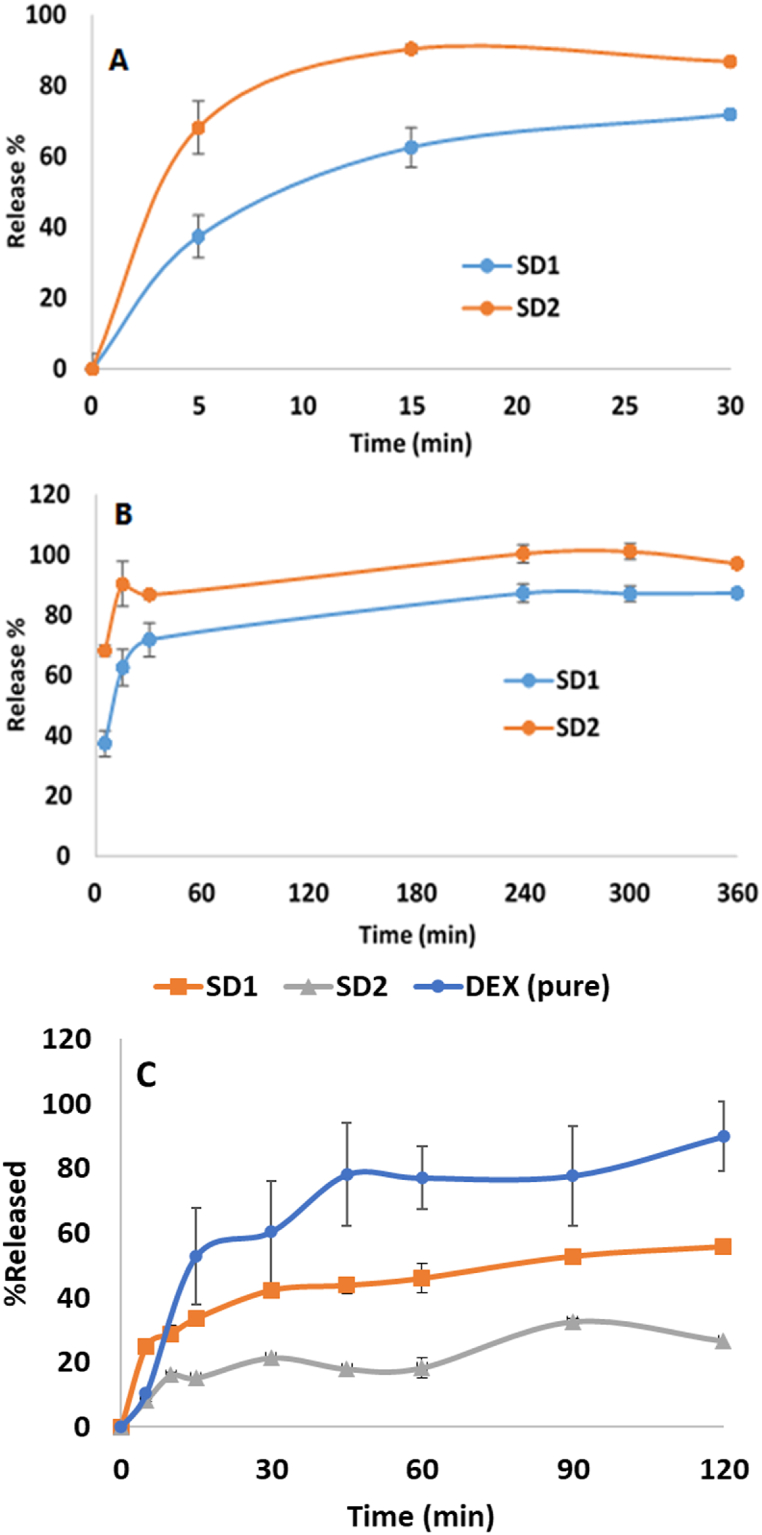
Dissolution profiles of SD1 and SD2 in simulated colon media (pH 7.4) at 37 **±** 1 °C for A) early stage release profile 30 min, B) 360 min and C) Dissolution profiles of DEX, SD1 and SD2 in acidic media (pH 1.2) at 37 ± 1 °C. Data are presented as mean ± SD (n = 3).

Fig. 5 shows the dissolution profiles of SD1 and SD2 in acidic media, along with pure DEX crystals (pH 1.2). The percentage of DEX released from formulation SD2 was significantly lower than either SD1 or pure DEX (*p* < 0.01). After 5 min, the release of DEX from SD1 was considerably higher compared to that from SD2 (25.04 vs. 8.28 %). Then, SD1 continued to exhibit a higher release at subsequent time points (10–60 min) compared to SD2. After 30 min, the percent release of DEX from SD1 and SD2 was 42.37 and 21.50 %, respectively. After 120 min, the release of SD1 continued to increase and be higher than that of SD2 (55.77 vs. 26.71 %). However, the United States Pharmacopeia (USP) does not mention any general methodology to study the release from colon-targeting dosage forms [60]. Our goal was to develop a colon-specific solid dispersion that delays drug release in the stomach but progressively releases the drug in the colon. The results obtained in the pH 1.2 release showed that both SD1 and SD2 displayed significant (*p* < 0.01) release control over pure DEX (DEX: 89 % at 120 min; SD1: 55 % at 120 min, SD2: 26 % at 120 min). Both formulations were also evaluated at colonic pH to validate our findings.

The dissolution of SD1 and SD2 was evaluated in colon-simulated medium (0.05 M phosphate buffer solution, pH 7.4) (Fig. 5A and B). Both SD1 and SD2 showed an increase in the release percentage over time. SD2 exhibited a higher release percentage than SD1 over the first 30 min (Fig. 5A). For example, the release percent of DEX from SD2 was significantly higher than that from SD1 (*p* < 0.01) after 5 min (68.24 vs. 37.42 %). During the first 15 min, DEX was gradually released from SD1 and SD2, and then the release reached a plateau of approximately 80 %. After 30 min, the release of SD2 was significantly higher (*p* < 0.05) than SD1 in the colon simulated medium. In colon-simulated media, the SD2 produced from EU S100 alone tends to release significantly more than the one produced from HPMC along with EU S100 (SD1). Several reasons may be responsible for this variation in drug release behavior, such as polymer properties and interaction between polymers.

Regarding polymer properties, EU S100 is a pH-sensitive polymer that dissolves at higher pH levels, which is generally found in the colon, but is insoluble in the gastric simulated fluid [24]. This characteristic makes it possible to target the colon specifically for drug release. On the other hand, the hydrophilic polymer HPMC might not exhibit the same pH-dependent release characteristic as EU S100 [61]. It might break down more easily in the stomach, resulting in a higher gastric release of the drug than EU S100.

Of the two formulations, formulation SD2 appears to be the more capable colon-targeting formulation as pharmacopoeial requirements recommend average drug release not exceed 20 % after 120 min at pH 1.2 simulated gastric fluids as shown in Fig. 5C [62]. Notably, the USP does not regard the 20 % drug release has a hard limit, but rather as a guideline with some acceptable tolerances This means that while the minimum requirement is 20 %, the actual release can be slightly higher or lower within a specified tolerance, ensuring that the formulation meets the pharmacopoeial standards without being too strict to accommodate the properties of different formulations [[63], [64], [65], [66]].

### 3.8 Solubility study

DEX exhibited poor aqueous solubility of 89 μg/mL, limiting its oral bioavailability [67]. It has been shown that the solubility of DEX enhanced upon dispersion in several carriers such as polyethylene glycol 6000 [68], propylene glycol [69], and chitosan [70]. In this study, the solubility of DEX significantly improved upon its dispersion in the polymeric carriers at 37 ± 1 and 25 ± 1 °C in PBS (pH = 7.4), compared to that of pure DEX (Table 2). While at acidic media (pH 1.2), both DEX and SD2 show significantly different solubility profiles. DEX exhibits significantly higher solubility (*P* < 0.05) compared to SD2 at biological temperature 37 °C as shown in Table 2. Specifically, at 37 ± 1 °C, the solubility of DEX is 895.13 ± 28.94 μg/mL, whereas the solubility of SD2 is considerably lower at 159.43 ± 7.30 μg/mL. This trend is consistent at 25 ± 1 °C as well. The results indicate that the solid dispersion formulation (SD2) enhances the solubility of dexamethasone compared to its free form (DEX) in basic media while in acidic media the solubility was significantly reduced by formulating it as solid dispersion with pH-sensitive carrier EU S100.

**Table 2:** The solubility of pure DEX and SD2 at 25 ± 1 and 37 ± 1 °C at acidic and basic pH (Mean ± SD.).

	Basic media (pH 7.4) (μg/mL)		Acidic media (pH 1.2) (μg/mL)	
	37 ± 1 °C	25 ± 1 °C	37 ± 1 °C	25 ± 1 °C
DEX	64.40 ± 0.00	53.65 ± 0.13	895.13 ± 28.94	886.66 ± 24.07
SD2	368.50 ± 1.17	100.36 ± 0.06	159.43 ± 7.30	164.24 ± 2.99

The improvement in solubility at basic conditions is attributed to the conversion of DEX from the crystalline to amorphous form upon its dissolving within the polymers, as confirmed by the DSC and PXRD results. In addition, the higher solubility of SD2 in PBS (pH 7.4), compared to pure DEX, is attributed to the utilization of the pH-sensitive carrier EU S100, which is recognized by its physical interaction with DEX molecules and its good solubility at pH > 7.0, used for colon-targeting [71]. Moreover, the solubility of DEX in SD2 was improved due to the high wettability of the amorphous state of SDs compared with pure DEX [72]. The incorporation of DEX into a solid dispersion decreased the particle size of the drug from a packed crystal to a molecular dispersion, thereby increasing the surface area available for dissolution, resulting in the observed enhanced dissolution rate [73]. This ultimately improves the dissolution rate and the bioavailability of poorly water-soluble drugs such as DEX [74,75].

## 4 Conclusion

Solid dispersion formulations, prepared by the solvent evaporation method, were successfully developed to enhance the solubility of DEX due to conversion into amorphous form, which was confirmed by DSC. SD2 which was prepared using EU S100 has the best enhancement in the release profile with a dissolution rate that reached more than 80 % within 30 min in the colon simulated media and less than 20 % in acidic media. The use of DEX-SDs was found to be a promising technique to enhance the solubility and dissolution of DEX which can impact its therapeutic efficacy. Future work should be designed to perform accelerated stability studies, permeability and in vivo studies for the selected formulation, SD2.

## Data availability

The data that has been used is confidential.

## Ethics approval and consent to participate

Not applicable.

## CRediT authorship contribution statement

**Ahlam Zaid Alkilani:** Writing – review & editing, Writing – original draft, Supervision, Resources, Project administration, Investigation, Conceptualization. **Sara Omar:** Writing – original draft, Methodology, Investigation, Formal analysis. **Jehad Nasereddin:** Writing – review & editing, Writing – original draft, Formal analysis, Conceptualization. **Rania Hamed:** Writing – review & editing, Data curation. **Rana Obeidat:** Writing – review & editing, Resources, Methodology.

## Declaration of competing interest

The authors declare that they have no known competing financial interests or personal relationships that could have appeared to influence the work reported in this paper.
