# Supplementary material for: Design of colon-targeted drug delivery of dexamethasone: Formulation and in vitro characterization of solid dispersions
Source: Heliyon. 2024 Jul 6;10(14):e34212. doi: 10.1016/j.heliyon.2024.e34212 (PMC11295959; doi:10.1016/j.heliyon.2024.e34212)
Supplement: Multimedia component 1 [file mmc1.docx]

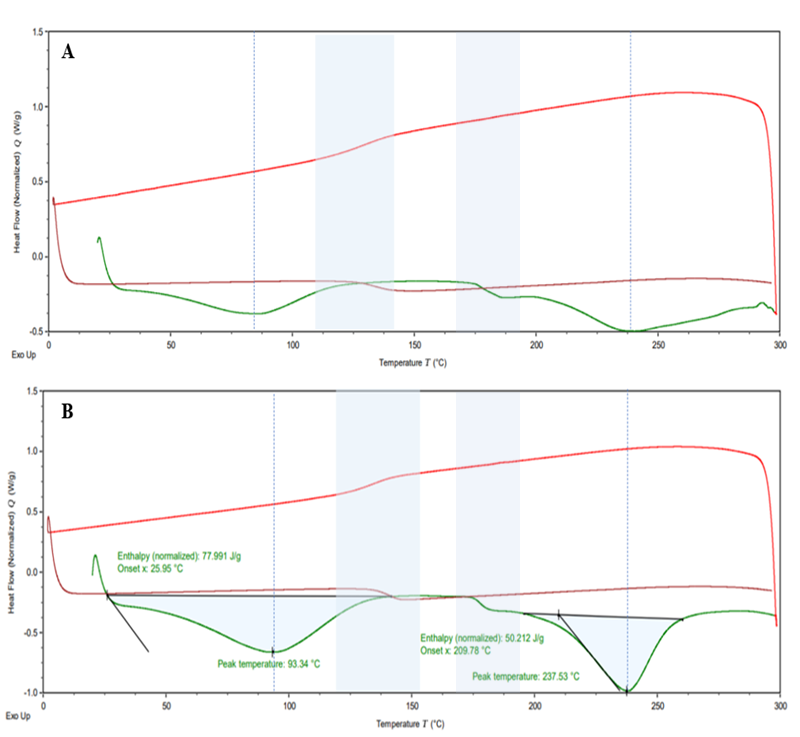


**Figure 1:** DSC thermogram of A: physical mixture of SD1 and B: physical mixture of SD2. In both thermograms, the plot in green represents the first heating cycle, and the plot in burgundy represents the second heating cycle.
